# Perspectives on health and well-being in human vulnerability

**DOI:** 10.3402/qhw.v11.31477

**Published:** 2016-05-10

**Authors:** Henrika Jormfeldt

**Affiliations:** School of Health and Welfare, University of Halmstad, Halmstad, Sweden, E-mail: Henrika.jormfeldt@hh.se

As a guest editor of the special edition of the *International Journal of Qualitative Studies on Health and Well-being* I would like to present seven designated articles. The current thematic cluster represents qualitative research illustrating the challenges in supporting the central aspects of health and well-being for individuals in various situations linked to human vulnerability. This theme cluster will provide the reader with a new and greater understanding of the inner meaning of vulnerability and greater insights into how health and well-being, achieved through different forms of support and care, can enhance empowerment in spite of obstacles such as illness, disease, impairment, old or young age, or gender. Different kinds of qualitative methodologies have been used to elucidate the phenomenon of vulnerability. Data collection procedures vary from qualitative interviews and diaries to focus group interviews. Qualitative content analysis, phenomenographical approach, and grounded theory are used to analyse the data in the different studies. The common denominator for the included articles is the commitment of the authors to impart knowledge in terms of greater understanding of the core aspects of health and well-being among humans in different vulnerable situations.

Vulnerability is a condition that is relevant for all human beings, but is especially actualized in connection with loneliness, injury, ill health and death. An overall responsibility for the professionals who take care of people in situations where they face vulnerability is thus to help and support the exposed individuals in attaining improved health and well-being. Professionals, such as psychologists, social workers and nurses from all the specialities, have a responsibility in their work to facilitate their clients’ empowerment processes. A prerequisite for them to be able to contribute with help and support in a constructive manner is knowledge about what support is needed for people in vulnerable situations. Participation in healthcare situations among children with juvenile idiopathic arthritis is one specific example where healthcare professionals need more knowledge from the perspective of the children. A constructivist grounded theory is used in order to understand the factors that influence the promotion of increased child participation in healthcare (Gilljam, Arvidsson, Nygren, & Svedberg, 2016). Previous research into the participation of children with juvenile idiopathic arthritis in healthcare situations most often focussed on the perspectives of the parents and healthcare professionals but not on the perspectives of the vulnerable children themselves. Adult patients with established rheumatoid arthritis are also vulnerable and they often experience that quality of life entails a constant balancing of lifestyle habits between the ideal situation (ideality) and the actual situation (reality) (Malm et al., 2016). The knowledge of how lifestyle habits for these patients influence their quality of life in terms of limitations, self-regulation, and companionship provides a further example of human vulnerability which is elucidated by means of a qualitative content analysis according to Graneheim and Lundman (2004). A woman in early labour represents another example of a vulnerable situation. Early labour is depicted as the very first phase of the labour process when professionals are generally deemed not to be needed (Carlsson, 2016). Women have reported that they are often left in a vulnerable situation in this phase, where their preferences are not always met and where they are not always included in the decision-making process. One core prerequisite for midwifes is thus knowledge about how to support women in early labour so that they feel that they are in a safe and secure place. A grounded theory approach was used to reveal a substantive theory of women needs.

In order to facilitate the recovery of women after a myocardial infarction, a qualitative content analysis was carried out (Wieslander, Mårtensson, Fridlund, & Svedberg, 2016). Findings suggest that both a preventive and promotive perspective should be taken into consideration in personal recovery among women after a myocardial infarction. Health promotion also plays an important role in the management of diabetes and chronic kidney disease in Vietnam, especially when the prevalence of the disease is rising there (Pham & Ziegert, 2016). To gain knowledge about the needs for health promotion among Vietnamese nurses a phenomenographical approach was adopted, which concluded that health promotion needs to be further integrated into the education if these vulnerable people are to receive adequate and appropriate support (Pham & Ziegert, 2016). Vulnerability among elders with a high fall risk in independent living was elucidated by using a classic grounded theory design showing that seniors were resolving their main concern of being able to carry on being themselves as they used to in their earlier lives by self-preservation strategies (Källstrand Eriksson, Hildingh, Buer, & Thulesius, 2016). The common issue in all of the presented articles highlights human vulnerability and the significance of the knowledge and ability needed to provide practical and emotional support in order to be able to help the individual verbalize his or her needs and desires.

These papers demonstrate that one essential and shared aspect of enhanced health and well-being can be understood as a relief from the position of vulnerability, regardless of its origin. Equality in decision-making processes is achieved through a genuine dialogue that aims to support the individual's personal resources and insights. The revealed knowledge about the nature of vulnerability may constitute a necessary prerequisite for the further development of our ability to reduce vulnerability and strengthen our capability for supporting coping strategies, such as decision making in accordance with the personal preferences of those in vulnerable situations. The presented articles indicate core aspects of perspectives on health and well-being when they are needed the most, such as in situations linked to human vulnerability. This theme cluster will provide the reader with new comprehension of the meaning of vulnerability and greater realisation into how intervntions to improve health and well-being can enhance empowerment regardless of illness, disease, impairment, old or young age, or gender.

*Henrika Jormfeldt*, RNT, PhD School of Health and Welfare University of Halmstad Halmstad, Sweden E-mail: Henrika.jormfeldt@hh.se
